# Multiple object tracking in the presence of a goal: Attentional anticipation and suppression

**DOI:** 10.1167/jov.24.5.10

**Published:** 2024-05-24

**Authors:** Andrea Frielink-Loing, Arno Koning, Rob van Lier

**Affiliations:** 1 Donders Institute for Brain, Cognition and Behaviour, Radboud University Nijmegen, Nijmegen, The Netherlands

**Keywords:** multiple object tracking, active MOT, attention

## Abstract

In previous studies, we found that tracking multiple objects involves anticipatory attention, especially in the linear direction, even when a target bounced against a wall. We also showed that active involvement, in which the wall was replaced by a controllable paddle, resulted in increased allocation of attention to the bounce direction. In the current experiments, we wanted to further investigate the potential influence of the valence of the heading of an object. In Experiments 1 and 2, participants were instructed to catch targets with a movable goal. In Experiment 3, participants were instructed to manipulate the permeability of a static wall in order to let targets either approach goals (i.e., green goals) or avoid goals (i.e., red goals). The results of Experiment 1 showed that probe detection ahead of a target that moved in the direction of the goal was higher as compared to probe detection in the direction of a no-goal area. Experiment 2 provided further evidence that the attentional highlighting found in the first experiment depends on the movement direction toward the goal. In Experiment 3, we found that not so much the positive (or neutral) valence (here, the green and no-goal areas) led to increased allocation of attention but rather a negative valence (here the red goals) led to a decreased allocation of attention.

## Introduction

Keeping track of moving visual objects in space over time is part of our daily tasks—for example, in doing sports, work, and traversing through traffic. Such multiple object tracking (MOT) tasks usually involve multiple objects of interest that must be tracked simultaneously. This means that we need to be able to divide our attention over several loci that often move independent from each other. Perhaps unsurprisingly, we are quite able to do so, even when the objects that have to be tracked look identical (Pylyshyn & Storm, 1988). However, there is a limit to how many objects we can keep track of (Alvarez & Franconeri, 2007; Cavanagh & Alvarez, 2005; Scholl, 2009). We previously proposed that the ability to track multiple objects is aided by not only attending to the current locations of objects but also taking into account their trajectories (Atsma, Koning, & van Lier, 2012; Frielink-Loing, Koning, & van Lier, 2017). Using probe detection, we showed that attention is indeed projected ahead of objects in anticipation of their future locations during MOT, especially when objects are tracked covertly (Frielink-Loing et al., 2017). Following the finding of Atsma et al. (2012) that attention does not “bounce” during passive viewing (i.e., that it does not shift to a new trajectory in anticipation of a predictable change of direction following a collision), we found that, when the task involved active interaction with the tracked objects, attention was shifted more toward the “bounce” path, when measured under the right conditions (Frielink-Loing, Koning, & van Lier, 2022). Therefore, in the present study, we investigated whether the context in the form of a goal is able to further direct attention toward the future trajectory of the object.

There are several ways in which allocation of attention can be measured and mapped. The original MOT task, developed by Pylyshyn and Storm (1988), has often been applied as a tool to investigate mechanisms of attention. In the original paradigm, a target flash identification task was used during the movement phase to measure how many targets participants could track simultaneously. Tracking performance is also often measured after each motion phase either by randomly highlighting an object and asking, “Was this a target or not?” (e.g., Yantis, 1992), or by requiring the participant to select all objects they believed were targets (e.g., Keane & Pylyshyn, 2006). In recent years, MOT has been used for closer investigation into the way attentional resources are distributed over the visual scene when observers perform a MOT task, not just between objects but also across the object surface and in the area surrounding them (e.g., Alvarez & Scholl, 2005; Atsma et al., 2012; Flombaum, Scholl, & Pylyshyn, 2008; Frielink-Loing et al., 2017; Frielink-Loing et al., 2022). This requires a different type of measurement than tracking performance.

For a more direct measure of where attentional resources are allocated during tracking, we make use of probe detection (Drew, McCollough, Horowitz, & Vogel, 2009; Posner, 1980). Specifically, we briefly present tiny dot probes at specific locations with respect to moving objects and compare probe detection rates between these locations to determine how attention is allocated during object tracking. This technique relies on the idea that attention lowers the threshold for stimulus detection (cf. Posner, 1980); thus, by measuring probe detection at multiple but specific locations with respect to a moving object, we can map the distribution of attention in and around a tracked object. In earlier studies, we used this technique to show that attention is distributed anisotropically around tracked objects (Atsma et al., 2012), specifically during covert tracking (Frielink-Loing et al., 2017), and that allocation of attention can be modulated by active interaction (Frielink-Loing et al., 2022). These findings suggest that attention is focused ahead of a moving target object, thereby appearing to take into account not just the current location of the object at each time point but also its predicted future trajectory.

In addition to active involvement, it seems likely that the relevance of the trajectory of an object also influences the allocation of attention; that is, naturally, if the trajectory of a target object is unpredictable (e.g., Howe & Holcombe, 2012), then using motion information to predict its future location (extrapolation) will not help tracking. Under such circumstances, one needs to attend to the current location of an object in order to be able to successfully track it. Conversely, when an object moves predictably (e.g., in a straight line), its future path can be anticipated (Atsma et al., 2012), although a computational investigation showed that extrapolation is not necessary for object tracking (Zhong, Ma, Wilson, Liu, & Flombaum, 2014). Moreover, and more relevant for the current study, what if the task is not only to keep track of objects but also to manipulate their movement such that they may approach, or avoid, a certain location or another object? After all, in real life, object tracking, generally, is a means to an end; we use it to be able to anticipate or to manipulate (objects in) our environment. In fact, a study by Thornton, Bülthoff, Horowitz, Rynning, and Lee (2014) showed that participants are very good at controlling object movements to avoid collisions during an interactive MOT task (iMOT) and can even control more objects than they can track during a regular MOT task.

Here, we present a form of interactive MOT employed in a paradigm where target objects must hit a goal in order to score points (Experiments 1 to 3), allowing us to investigate the distribution of attention in a new context. Additionally, we investigate the influence of goal valence (i.e., whether a goal should be approached or avoided; Renton, Painter, & Mattingley, 2019) on the allocation of attention around tracked objects (Experiment 3). In Experiment 1, participants performed a MOT task where they controlled a movable “goal” with which they were instructed to deflect (or “catch”) tracked objects. During the MOT task, as objects collided with a static wall at the center of the screen, a probe could appear either along the extrapolation of its pre-collision path (where the object would never go, because it always bounced off the wall) or along its post-collision path (cf. Atsma et al., 2012). We compare probe detection rates for these two probe locations (the so-called linear and bounce probes) in situations when the target object was moving toward the goal and when it was moving away from the goal. We expected to see enhanced attention anticipating an imminent collision between the object and the goal, measured as a relatively high detection rate for bounce probes compared to linear probes as the object moved toward the goal. No such difference in probe detection rates is expected when a tracked object is moving away from the goal.

In Experiment 2, the goal area was confined to a smaller part of the screen, which allowed us to distinguish whether proximity between the probe and the goal, or anticipation of scoring a point, could account for the enhanced probe detection for the “goal” probes in Experiment 1. In a third experiment, the goals were fixed in the corners of the display, and the participants were given control over the permeability of the static wall in the center of the screen (i.e., whether objects would bounce off or move through it). In the first two experiments, where objects always bounced, only bounce probes would appear in an actual future location of the object, but this third experiment allowed for investigation of the distribution of attention around a tracked object where both linear probes and bounce probes could appear in the future location of the object. We again expected to find enhanced allocation of attention along the path of an object moving toward a positive reward goal. Additionally, we expected to see a suppression of probes along the path between the object and a negative reward goal.

## Experiment 1

### Method

#### Participants

Thirty-two participants (27 females, five males; 23 ± 3 years old) took part in this experiment and received payment or course credit for their participation. Twenty-seven participants were right-handed. All reported to have normal or corrected-to-normal vision and were naïve with respect to the nature and objective of the study. Procedures were in accordance with the tenets of the Declaration of Helsinki, and all participants gave written informed consent before the start of the experiment.

#### Stimuli and design

**Figure 1.**
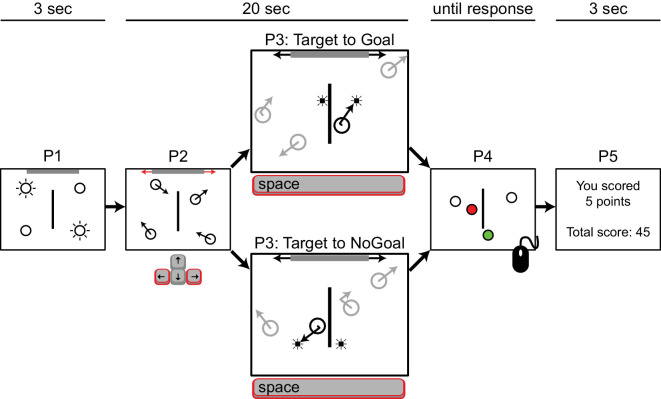
Timeline for Experiment 1. Each trial started with identification of the targets that had to be tracked by having them blink on and off for 3 seconds (panel P1). The goal (gray horizontal bar) appeared at either the top or bottom of the screen (randomly chosen at the start of each trial). Next, targets and distractors started moving while participants controlled the movable goal to intercept the targets by pressing the left and right arrow keys on a standard keyboard (panel P2). Whenever a target hit the vertical wall at the center of the screen, a probe could appear along the linear trajectory of the target, on the bounce trajectory (panel P3) or at a random location within the bounding box (see main text). Depending on the direction of the target with respect to the location of the goal, these probes were marked as goal probes (panel P3, top) or no goal probes (panel P3, bottom). At the end of the trial, participants were asked to identify the targets using the mouse (panel P4) and received feedback on the number of interceptions they made (panel P5).

A white rectangle subtending 25° × 20° was presented centrally on a black background, acting as a bounding box. On each trial, four identical objects were presented, two targets and two distractors. Objects were black circular outlines, 2° in diameter, and were randomly assigned to a corner of the bounding box (Figure 1, panel P1). A black vertical wall, 0.1° wide and 10° high, was presented at the center of the screen. A gray horizontal bar subtending 12.5° × 0.4° appeared centered along either the top or bottom wall of the bounding box and served as the goal in the experiment. Whether the goal appeared at the top or the bottom of the bounding box was randomly determined at the beginning of each trial.

At the start of each trial, the two target objects blinked on and off for 3 seconds to indicate that they were the objects the participant should keep track of (Figure 1, panel P1). Next, all four objects started moving in randomly generated directions for a duration of 20 seconds at a constant speed of 8°/s (Figure 1, panel P2). Upon encountering one of the four edges of the bounding box, the wall in the middle of the screen, or the goal, objects would bounce off naturally (angle of reflection equals angle of incidence as measured to the normal).

During the movement phase of a trial, participants could move the goal left or right by pressing the left or right arrow keys on a standard QWERTY keyboard. The bar moved at a constant speed of 16°/s left or right while the arrow key remained pressed, and its movement was restricted by the edges of the bounding box. Whenever a collision occurred between a target object and the vertical wall at the center of the screen, one of four possible experimental events could occur: a probe was shown along the bounce trajectory of the object (Bounce probe, 30% of events), a probe was shown along the linear extrapolation of the heading direction of the object before the collision (Linear probe, 30% of events), a probe was shown at a random location within the bounding box (Open Space probe, 20% of events), or no probe was shown (No Probe event, 20% of events). Bounce and Linear probes (for examples, see Figure 1, panel P3) could appear at one of three distances (2.4°, 3.2°, or 4.0°) from the center of an object. Probes were presented for 50 ms and moved along with the probed target to maintain their distance with respect to the object.

The Bounce and Linear probes were additionally categorized as Goal or No Goal events, depending on whether the target object that triggered the event was at that moment moving towards (Goal) or away (No Goal) from the horizontal wall containing the goal. Goal probes (bounce or linear) thus always appeared between the target object and the goal (in Figure 1, panel P3, top); No Goal probes (bounce or linear) thus always appeared between the target object and the horizontal wall not containing the goal (in Figure 1, panel P3, bottom). In case of a Goal event, we also recorded whether the target subsequently reached the goal (hit) or not (miss).

#### Procedure

Participants were seated in front of a liquid-crystal display monitor (resolution of 1920 × 1080 pixels and refresh rate of 120 Hz), and they operated a standard QWERTY keyboard and computer mouse. Participants were seated on a fixed chair approximately 60 cm from the screen. Stimuli were created and presented with PsychoPy 1.90.2 (Peirce, 2007; Peirce, 2009), running on a Dell Precision T3610 computer (Dell, Round Rock, TX) with Windows 7 (Microsoft, Redmond, WA).

At the start of the experiment, participants received instructions to track two out of four identical objects, which were indicated at the beginning of each trial, and to deflect the two target objects as often as possible with the movable goal. They were told that they would receive points each time they “caught” a target object, and at the end of each trial their total score would be presented (Figure 1, panel P5). Participants were also instructed to press the space bar each time they detected a small, brief dot probe on the screen. Responses to probes within 1000 ms after presentation were recorded. If no response was given within that time window, the probe was considered undetected. At the end of the movement phase of each trial, participants were asked to identify both targets using the mouse (Figure 1, panel P4). To provide feedback on their tracking performance, correctly identified targets turned green and distractors turned red when selected.

All participants first performed five practice trials of 20 seconds, followed by as many trials as necessary to obtain measurements for 400 events (320 Probe events and 80 No Probe events). Participants performed 95 trials on average (minimum 90, maximum 100), with the exception of two participants who performed 54 and 88 trials, respectively, due to technical problems causing the experiment to stop prematurely. The experiment lasted approximately 50 minutes, depending on the number of trials needed to complete data collection, including a break after every fifth trial.

### Results

Overall tracking performance was determined for each participant by calculating the average number of correctly identified targets over all trials. Mean tracking performance was moderate to high for most participants (*M* = 66%; *SD* = 14%; range, 47%–92%). Only events triggered by targets that were correctly identified at the end of a trial were considered for analysis to ensure that participants attended to the correct object during probe presentation. As a result, 144 out of 400 events were discarded on average per participant. Average probe detection rates were determined based on the responses to the remaining events. Only button presses that occurred within 1000 ms after event onset were counted as a hit (cf. Flombaum et al., 2008). Participants could be excluded for not seeing probes (less than 5% total probe detection for probe events relevant for analysis), high false alarm rate (measured as outliers), and low tracking performance (below or at chance level, 50%). Eight participants were excluded from analysis because their total probe detection rate for the Bounce and Linear probe events was lower than 5%.

**Figure 2.**
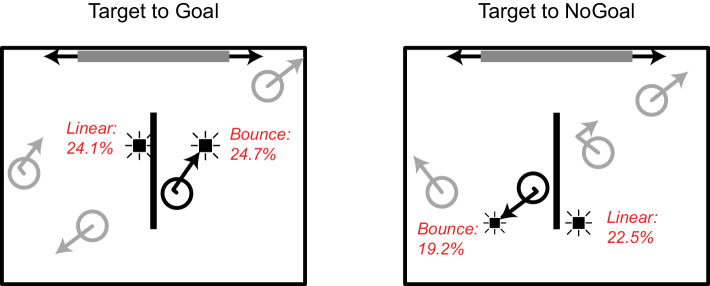
Mean probe detection rates superimposed on the situation sketch from Figure 1 (panel P3). Probe sizes represent their relative detection rate and do not reflect presentation size during the experiment.

For the remaining 24 participants, overall probe detection (i.e., responses to Probe events) was low to moderate (*M* = 19%; *SD* = 9.5%; range, 5%–45%). False alarm rates ranged from low to high (*M* = 5.3%; *SD* = 5.0%; range, 0%–17%), with no statistical outliers (the criterion for an outlier used here is that its value falls above the third quartile + 1.5 times the interquartile distance). Table 1 and Figure 2 show detection rates for the Bounce and Linear probes. Note that, because target objects that were heading toward the wall with the goal were almost always caught by the participant, we only considered probes triggered by target objects that were moving toward and eventually hit the goal (Goal events) and probe events triggered by target objects that were moving toward the opposite wall (No Goal events). Finally, due to data sparsity, we decided to collapse the results over probing distance for further statistical analyses.

**Table 1.** Experiment 1 probe detection results. Mean probe detection rates are expressed as percent (*SE*) based on 24 participants.

	Goal	No goal
Bounce probe	24.7 (2.6)	19.2 (2.5)
Linear probe	24.1 (2.9)	22.5 (2.7)

#### Probe detection

A 2 × 2 repeated-measures analysis of variance (ANOVA) with probe type (Bounce, Linear) and object heading (Goal, No Goal) as the within-subject factors and probe detection rate as the dependent variable revealed a significant main effect of object heading, *F*(1, 23) = 5.87, *p* = 0.024, η^2^ = 0.203, 1 – β = 0.641, with higher detection rates for probes triggered by target objects moving toward the goal than by target objects moving away from the goal (*M*_Goal_ = 24.4%, *M*_NoGoal_ = 20.9%). There was no main effect of probe type (*F* > 1), and no significant interaction between probe type and object heading (*F* = 2.33, *p* = 0.141).

### Discussion

For this first experiment, we used an interactive form of MOT including a goal to investigate whether the distribution of attention around a tracked object would differ when the tracked object moved toward the side where the movable goal was located compared to when it moved away from the goal. We indeed found a main effect of object heading, as probes were detected more often when a tracked object was moving toward the goal than when a tracked object was moving away from the goal, which shows that the presence of a goal does modulate the distribution of attention around tracked objects. We did not find a main effect of the type of probe (bounce vs. linear) nor an interaction effect between object heading (toward a goal vs. away from a goal) and the type of probe (bounce vs. linear). This means that here attention was enhanced when a probe was presented in the area between the tracked object and the goal, irrespective of whether the probe appeared in the bounce direction of the tracked object or along its linear direction.

The current study expands on research by Atsma et al. (2012), who found that attention does not shift toward the post-bounce trajectory when an object is colliding with an obstacle. In this first experiment, attention was rather equally distributed over the linear and bounce trajectories. The results show that attention is generally enhanced when a tracked object approaches a goal compared to when a tracked object does not approach a goal. Still, this can be interpreted in two ways: either the object moving toward the goal side of the box (top/bottom) enhances attention or the presence of the goal itself does. In order to distinguish between these two possible explanations, we performed a second experiment.

## Experiment 2

**Figure 3.**
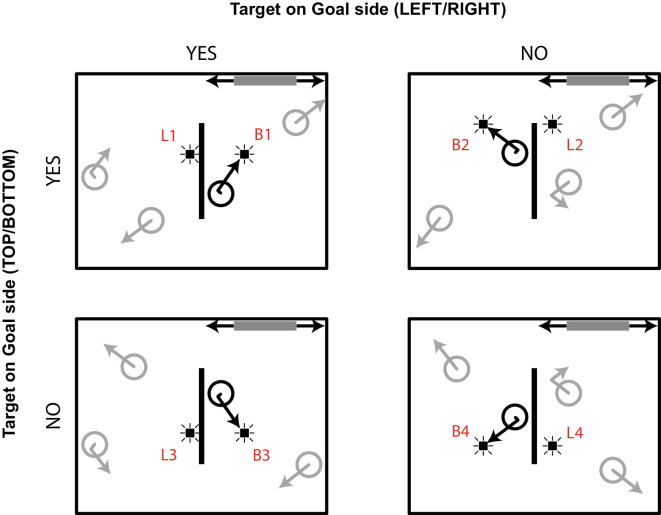
Probe types in Experiment 2 for probe events triggered by target objects moving toward the horizontal wall containing the goal. For illustration purposes, the goal is restricted to the upper right corner of the bounding box.

We adapted the task from Experiment 1 such that the goal could only move along half the width of the bounding box, essentially allowing participants to only deflect objects on either the right or left side of the screen (see Figure 3). We expected to see one of two possible outcomes: (a) both Bounce and Linear probes presented when an object bounces off the central wall heading toward the goal (e.g., toward the top-right quadrant; see probes B1 and L1 in Figure 3) are detected better than probes presented when the object bounces on the other side (e.g., toward the top-left quadrant; see probes B2 and L2 in Figure 3), which would indicate a rather object-centered enhancement of attention, versus (b) probes presented in the quadrant where the goal is located (B1 and L2) are detected better compared to probes presented in the quadrant where the goal is not located (B2 and L1), which would imply a more goal-centered enhancement of attention.

### Method

#### Participants

Twenty-seven new participants (21 females, five males, one other; 25 ± 12 years old) took part in this experiment and received payment or course credit for participation. All reported to have normal or corrected-to-normal vision and were naïve with respect to the nature and objective of the study. Procedures were in accordance with the tenets of the Declaration of Helsinki, and all participants gave written informed consent before the start of the experiment.

#### Stimuli and design

All stimuli were identical to those presented in Experiment 1, except for the goal, which now subtended a quarter of the width of the bounding box (6.25°). The goal could appear along either the top or bottom wall of the bounding box, centered on either the right or left half of the wall. The location of the goal was determined at random at the start of each trial by the PsychoPy program and was recorded along with the data that were collected. The movement of the goal was restricted to one-half of the bounding box by the program.

#### Procedure

The procedure for this experiment was nearly identical to the procedure for Experiment 1, with the additional instruction that the goal could only be moved along one-half of the wall of the bounding box. This also meant that, if an object could not be caught due to this restriction, it would not be counted as a miss. Participants performed 97 trials on average (minimum 92, maximum 106). The experiment lasted approximately 64 minutes, depending on the number of trials needed to complete data collection, including a break after every fifth trial.

### Results

**Figure 4.**
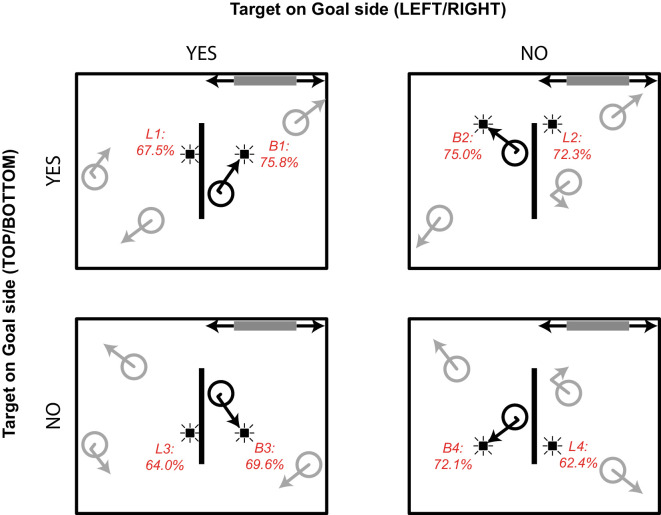
Mean probe detection rates superimposed on the situation sketch from Figure 3.

Overall tracking performance was determined for each participant by calculating the average number of correctly identified targets over all trials. Mean tracking performance was moderate to high for most participants (*M* = 75%; *SD* = 12%; range, 50%–94%). Only events triggered by targets that were correctly identified at the end of a trial were considered for analysis to ensure that participants attended to the correct object during probe presentation. As a result, 99 out of 400 events were discarded on average per participant. Average probe detection rates were determined based on the responses to the remaining events. Only button presses that occurred within 1000 ms after event onset were counted as a hit (cf. Flombaum et al., 2008). Exclusion criteria can be found in the Results section of Experiment 1. During a session with one participant, the experiment stopped prematurely due to a technical error, and the results for this participant were lost. Three participants were excluded from analysis because they formed an outlier on false-alarm rate (for the exclusion criterion, see the Results section in Experiment 1) or because their tracking performance was at chance level (∼50%). For the remaining 23 participants, overall probe detection (i.e., responses to Probe events) was high (*M* = 65%; *SD* = 13.9%; range, 23%–89%). False-alarm rates were low (*M* = 1.3%; *SD* = 1.5%; range, 0%–5%). Table 2 and Figure 4 show detection rates for the Bounce and Linear probes.

**Table 2.** Experiment 2 probe detection results. Mean probe detection rates are expressed as percent (*SE*) based on 24 participants. Note that goal side refers to the location of the tracked object, not the probe.

		Goal side (Left/Right)	
		Yes	No
Goal side (Left/Right)			
Yes	Bounce probe	75.8 (3.2)	75.0 (3.2)
	Linear probe	67.5 (3.4)	72.3 (3.2)
No	Bounce probe	69.6 (3.8)	72.1 (3.4)
	Linear probe	64.0 (2.9)	62.4 (3.1)

#### Probe detection

In order to test our two hypotheses, we performed a 2 × 2 repeated-measures ANOVA with object near goal (yes, no) and probe near goal (yes, no) as within-subject factors and probe detection rate as the dependent variable. Specifically, looking at Figure 4, probes B1 and L1 belonged to the target at the same side of the goal (i.e., object near goal: yes), whereas probes B2 and L2 belonged to the target on the opposite side of the goal (i.e., object near goal: no). Similarly, in Figure 4, probes B1 and L2 were presented at the same side of the goal (probe near goal: yes), whereas probes B2 and L1 were presented at the opposite side of the goal (i.e., probe near goal: no). The analysis revealed a main effect of probe near goal, *F*(1, 22) = 4.61, *p* = 0.043, η^2^ = 0.173, 1 – β = 0.537, with higher detection rates for probes that appeared in the goal corner of the bounding box. There was also a significant interaction between object near goal and probe near goal, *F*(1, 22) = 11.06, *p* = 0.003, η^2^ = 0.335, 1 – β = 0.888. As can be seen in Figure 4 (top row), mean probe detection was lowest when the object approached the central wall on the side where the goal was located, but the probe was not near the goal (see L1 in Figure 4)—that is, a linear probe irrelevant to the task. In contrast, both bounce probes (B1 and B2 in Figure 4) and the linear probe *near* the goal (L2 in Figure 4) yielded similar detection rates. There was no significant main effect of object near goal (*F* = 2.54, *p* = 0.125).

### Discussion

This second experiment was created to investigate the relative contributions of attention to the tracked object (target) and attention to the part of the bounding box containing the goal, which together might have contributed to the main effect of object heading in Experiment 1. That is, in Experiment 2, with the same vertical wall in the center of the screen, but now restricting the range of the moveable goal to the left or right side of the display, we could additionally distinguish the situation where the tracked object was heading toward the wall where the goal was located, as well as it being on the side of the vertical wall where the goal was located, from the situation where the tracked object was heading toward the wall where the goal was located but on the other side of the vertical wall (to where the goal could not be moved). We expected one of two outcomes: (a) a rather object-centered enhancement of attention, reflected by a higher detection of probes triggered by an object moving on the side where the goal could catch it (B1 and L1 in Figure 3); or (b) a more goal-centered enhancement where probes presented in the “quadrant” of the display of the goal are detected better (B1 and L2 in Figure 3).

What we can conclude from Experiments 1 and 2 so far is that goals appear to have an enhancing effect on probe detection. However, the interaction effect we found here suggests that in this second experiment there was neither an overall object-centered enhancement of attention nor an overall goal-centered enhancement of attention. That is, the results appear to be driven mainly by the lower probe detection for the condition in which the object was near the goal while the probe was presented on the opposite side (i.e., L1 in Figures 3 and 4). Or, stating it otherwise, the actual bouncing path of the object toward the goal apparently attenuated attention to the no-goal side. The following aspects should be noted here, which we will deal with in Experiment 3. First, whereas bounce probes are always on the same side of the vertical wall as the goal, this is not the case for linear probes. Naturally, this imbalance is the result of the targets always bouncing and never “going through” the wall. Experiment 3 was set up to deal with this imbalance by allowing the targets to also go through the wall. More specifically, wall permeability could be manipulated by the participant in Experiment 3. Second, following the influence of action on probe detection (Frielink-Loing et al., 2022), it seems plausible that this imbalance is further strengthened by the action that has to be taken on the goal. Thus, in Experiment 3, action and goals are decoupled; that is, just as in Frielink-Loing et al. (2022), we introduced a task in which the action occurs in the area of the central wall. The goals are still located in the corners of the box, but they will be static (although changing position from trial to trial). The action will be such that both Bounce probes and Linear probes appear at the quadrant toward which the object might be heading. The trick is to introduce an action task in which the wall can be made permeable by the participant when needed, triggered by the positioning of the goals that have to be reached, or avoided.

## Experiment 3

In this third experiment, we again employed a MOT paradigm in which two target objects among two distractor objects had to be tracked while at the same time probes must be detected. Instead of moving the goal around on the screen, attracting attention, the goal was now fixed in one of the corners of the bounding box. The vertical wall in the middle of the bounding box was extended to span the entire height of the box and could be made permeable or solid by the participant by pressing or releasing a button. To make the task as challenging as the previous tasks, a second “goal” was added that was to be avoided instead of hit. When a target object hit a positive goal, a point was gained; when a target object would hit a negative goal, a point was subtracted. For example, when a target object approached the central vertical wall as it was heading in the direction of a positive goal, it paid for the participant to make the wall permeable, allowing the object to continue along its path and hit the positive goal, resulting in a scored point. Similarly, to provide another example, when a target object approached the central wall as it was heading in the direction of a negative goal, it paid for the participant to make the wall solid, allowing the object to bounce off to avoid the negative goal, thereby avoiding the subtraction of a point. Within this design, both bounce probes and linear probes can appear in the direction of actual movement of the tracked object following an action of the participant (as they can make the wall permeable or solid). In sum, this task allows us to investigate the distribution of attention around tracked objects when they must be made to approach certain areas of the display and avoid others, by either moving through or bouncing off a wall as manipulated by the participant. We expect that probe detection is modulated by goal presence. More in particular, probe detection for objects moving toward a positive goal may be higher than toward a negative goal. The addition of neutral goals may then act as a baseline condition to decide whether it is in fact an enhancement or rather a suppression effect.

### Method

#### Participants

Thirty-one new participants (25 females, six males; 24 ± 5 years old) took part in this third experiment and received payment or course credit for participation. All reported to have normal or corrected-to-normal vision and were naïve with respect to the nature and objective of the study. Procedures were in accordance with the tenets of the Declaration of Helsinki, and all participants gave written informed consent before the start of the experiment.

#### Stimuli and design

**Figure 5.**
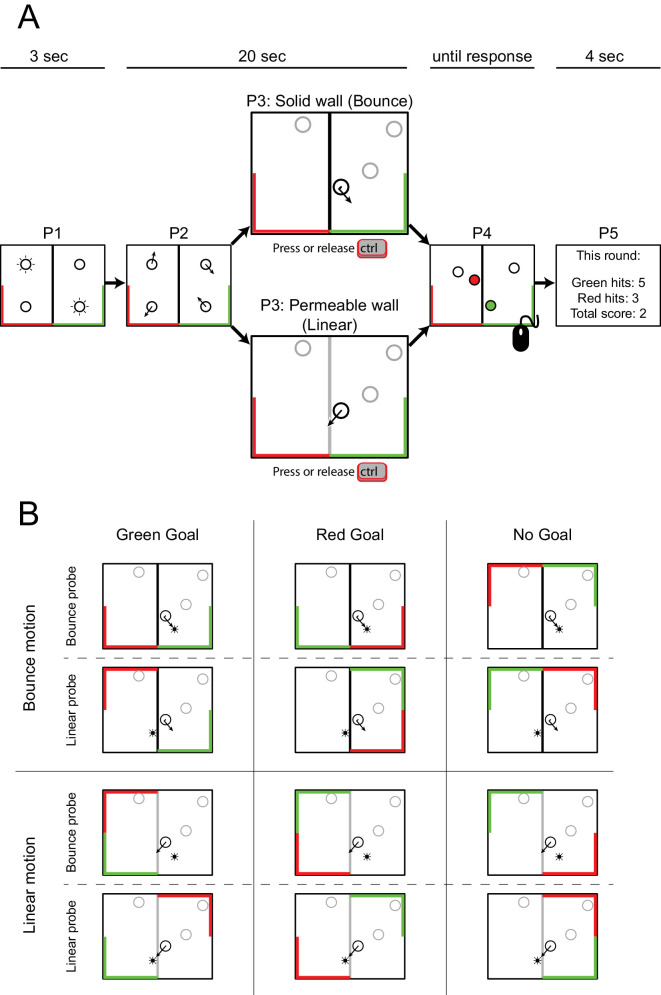
Illustration of the timeline (**A**) and conditions (**B**) for Experiment 3. (**A**) Each trial started with an identification of the targets to be tracked (panel P1). Next, targets and distractors started moving (panel P2). Participants controlled the permeability of the centralized wall by pressing or releasing the right Ctrl key on a standard keyboard in order to be able to manipulate the targets and let them reach the green goal but avoid the red goal (bottom panel in panel P3 shows the permeable wall as gray for illustrative purposes; the wall did not change color during the experiment). At the end of the trial, participants were asked to identify the targets using the mouse (panel P4). Panel P5 shows example feedback on the number of times targets had hit the green and red goals and also the total score (i.e., subtracting the red hits from the green hits). (**B**) For each trial the positions of the green and red goals were randomly assigned to two corners of the box. With the remaining two corners of the box not being assigned a color (i.e., the neutral goals), this resulted in the 12 shown displays, each with a unique goal configuration. Next, the participant could manipulate the permeability of the centralized wall by pressing and releasing a button, shown as black (solid) or gray (permeable) in the figure (note that, in the experiment, the wall remained black throughout). The top two rows show the centralized wall in black, indicating that whenever an object would collide with the centralized wall the object would bounce off of it. The bottom two rows show the centralized wall in gray, indicating that whenever an object would collide with the centralized wall the object would continue along its motion path. Finally, the (non-)permeability of the centralized wall and the locations of a presented probe (left or right of the wall) and an object that is about to collide with it (coming from the left or the right) resulted in probes being labeled as either a Bounce probe or a Linear probe, as shown in the figure. In all displays, the objects not colliding with the wall are shown in gray for illustrative purposes.

The bounding box and circular objects were identical to those presented in Experiments 1 and 2. A black vertical wall, 0.2° wide, divided the bounding box into two compartments. One green goal (positive) and one red goal (negative), each subtending 12.5° along a horizontal border and 10° along an adjacent vertical border, were randomly assigned by the computer program to a corner of the bounding box on every trial (Figure 5A, panel P1). The goals could be assigned to any corner of the box, as long as they did not overlap. Figure 5B depicts all possible positions of the goal and no-goal areas and shows (non-exhaustive) examples of Bounce and Linear probe events as the object moves toward the green goal, red goal, or no-goal area. Depending on an action of the participant (who could decide to make the wall permeable or solid when a target was about to hit the wall by pressing or releasing a single button), the object continued along its motion path and thus went “through the wall” (linear motion), or the object bounced off the wall naturally (bounce motion), with the angle of reflection being equal to the angle of incidence as measured to the normal.

At the start of each trial, two target objects blinked on and off for 3 seconds to indicate that they were the objects the participant should keep track of (Figure 5A, panel P1). Next, all four objects started moving in randomly generated directions for a duration of 20 seconds at a constant speed of 8°/s (Figure 5A, panel P2). Upon encountering one of the four edges of the bounding box or the (solid) wall in the middle of the screen, objects would bounce off naturally (angle of reflection equals angle of incidence as measured to the normal).

During the movement phase of a trial, participants could control the permeability of the vertical wall using the right Ctrl key on a standard QWERTY keyboard. How wall permeability could be toggled was counterbalanced, with half of participants having to press the Ctrl key and the other half of participants having to release the Ctrl key to let an object pass through. Each time a target object collided with the central wall, one of four experimental events could occur similar to Experiments 1 and 2 (i.e., Bounce, Linear, or Open Space Probe event or No Probe event). The chance of each event occurring was the same as in Experiments 1 and 2. Bounce and Linear probes could appear at the same three distances as used before (2.4°, 3.2°, or 4.0° from object center), and probes were again presented for 50 ms, moving along with the probed target to maintain their distance with respect to the object. For each event, the motion of the target was saved (through the wall or bounced off), as well as the presence and type of goal in both the corner the object approached and the corner the object avoided (green, red, or no goal) (Figure 5B).

#### Procedure

At the start of the experiment, participants received instructions to track two out of four identical objects, which were indicated at the beginning of each trial. They were informed that their task was to maximize their score by letting the two targets hit the green goal and avoid the red goal as often as possible by manipulating the permeability of the wall. No instructions were given regarding the distractor objects. Participants were informed that they would receive one point for each time a target hit the green goal and that they would lose a point each time a target hit the red goal. At the end of each trial their scores were presented onscreen (Figure 5A, panel P5). Crucially, participants were also instructed to press the space bar each time they detected a small, brief dot probe on the screen. Responses to probes within 1000 ms after presentation were recorded. If no response was given within that time window, the probe was considered undetected. At the end of the movement phase of each trial, participants were asked to identify both targets using the mouse (Figure 5A, panel P4). To provide feedback on their tracking performance, correctly identified targets turned green and distractors turned red when selected.

All participants received detailed instructions onscreen, as well as a brief demo explaining wall permeability and probe presentation. They then performed five practice trials of 20 seconds, followed by as many trials as necessary to obtain measurements for 600 events (480 Probe and 120 No Probe events). Thus, participants performed 75 trials on average (minimum 50, maximum 85). The experiment lasted approximately 45 minutes, depending on the number of trials needed to complete data collection, including a break after every fifth trial.

### Results

Overall tracking performance was determined for each participant by calculating the average number of correctly identified targets over all trials. Mean tracking performance was high for most participants (*M* = 82%; *SD* = 15%; range, 49%–97%). Only events that belonged to correctly identified targets were considered for analysis to ensure that participants attended to the correct object during probe presentation. As a result, 193 out of 600 events were discarded on average per participant. Average probe detection rates were determined based on the responses to the remaining events. Only button presses that occurred within 1000 ms after event onset were counted as a hit (cf. Flombaum et al., 2008). Eight participants were excluded from analysis because their total probe detection rate for Bounce and Linear probe events was lower than 5% or because their tracking performance was at chance level.

For the remaining 23 participants, overall probe detection (i.e., responses to all Probe events, including Open Space probe events) was low to moderate (*M* = 24%; SD = 14.4%; range, 4%–54%). False alarm rates were low (*M* = 1.6%; *SD* = 1.8%; range, 0%–7%). We collapsed the results over probing distance for further analyses to overcome data sparsity. Table 3 shows the mean detection rates for all probes, split over the approached goal (green, red, or no goal) and the type of motion (bounce or linear).

**Table 3.** Experiment 3 probe detection results. Mean probe detection rates are expressed as percent (*SE*) based on 23 participants. Results are split over the type of goal that was approached (green, red, or no goal) and which interaction with the wall preceded it (bounce or linear motion).

	Green goal	Red goal	No goal
Bounce motion			
Bounce probe	32.8 (3.4)	18.2 (5.6)	33.7 (3.8)
Linear probe	33.8 (3.7)	22.5 (4.9)	30.0 (3.4)
Linear motion			
Bounce probe	16.2 (4.5)	6.0 (2.7)	15.6 (3.9)
Linear probe	16.6 (4.7)	11.8 (4.2)	14.4 (3.7)

#### Probe detection

We performed a 2 × 2 × 3 repeated-measures ANOVA with move type (bounce, linear), probe type (Bounce, Linear), and goal type (green, no goal, red) as within-subject factors. Due to missing values (specifically for red goal events, as participants avoided hitting these goals), data of only 17 participants could be analyzed. There was a significant main effect of move type, *F*(1, 16) = 25.30, *p* < 0.001, η^2^ = 0.613, with a higher detection rate for probes presented when a target bounced off the wall compared to when a target went through the wall. We also found a significant effect of goal type, *F*(2, 15) = 6.38, *p* = 0.010, η^2^ = 0.458. Probes that were presented when a target moved toward a goal with a negative valence (i.e., the red goal) were detected significantly less compared with probes presented when a target moved toward either a positive goal (green) or a neutral goal (no goal). No other significant effects were found (all *p* > 0.2).

#### Valence

**Figure 6.**
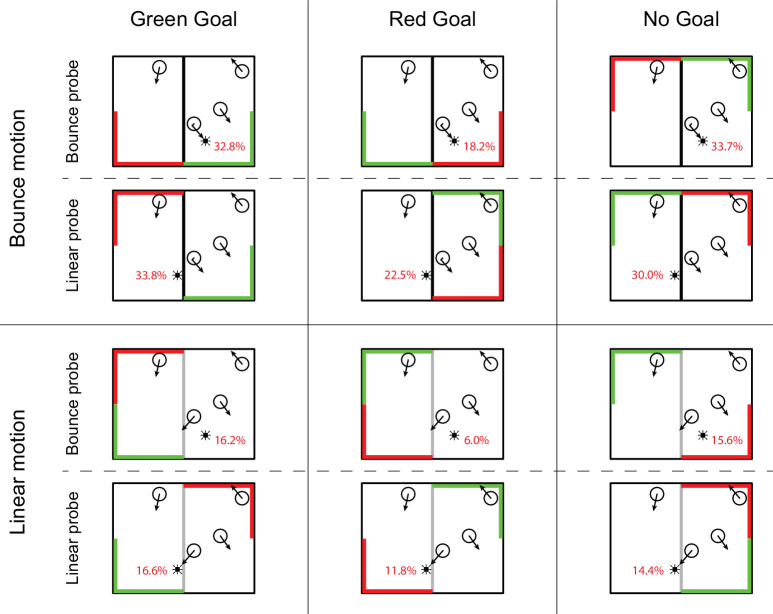
Results of Experiment 3 superimposed on the situation sketch from Figure 5. Note that these situation sketches are not exhaustive; that is, there were multiple goal configurations possible for each item in the table. For example, in the bottom-right corner, the probed object was let through the wall to reach a no-goal area, thereby missing the green goal. Alternatively, the two goals might be switched and the object could have avoided the red goal. For the results depicted here, these two examples would describe the same situation.

In the final analyses we also take into account the effect of avoiding (or missing) a (no-)goal area as a result of controlling the permeability of the central wall. That is, we performed an additional analysis in which we considered not only the properties of the quadrant of the box which the target was heading toward (green goal, red goal, or no goal) but also the properties of the quadrant at the same side (top or bottom) which the target then was not heading toward as a result of the decision and the corresponding action of the participant to make the wall permeable or not. As an example, a participant might choose to let a target bounce off the wall to make it head toward a no-goal area to avoid it hitting the red goal. Another possible situation was that a target was accidentally allowed through the wall toward the red goal, but it would have otherwise reached the green goal if it had bounced (although this situation occurred far less than the first example). Table 4 shows how the combinations of reached and avoided goal (or no-goal) area could be classified in terms of reward and punishment. The various combinations suggest various strengths regarding approaching or avoiding a specific direction and the attentional resources ahead of the target in that direction (as reflected by the probe detection rate). We labeled each combination with a specific valence value. So, if a target (as a result of the permeability response of the participant) headed toward a green goal to avoid a no-goal (as in Figure 6, bottom left), the probe detection in that direction was labeled with a single plus (+), and similarly so when the target headed toward a no-goal area to avoid a red goal (as in Figure 6, third row right). When a target headed toward a green goal to avoid a red goal (as in Figure 6, top left) a double plus (++) was assigned to that cell. The cells with a negative valence were defined in a similar way. In the situation where a target headed toward a red goal to “avoid” a no-goal area (by a wrong permeability response as in Figure 6, bottom center) a single minus (–) label was assigned, and similarly so when the target headed toward a no-goal area to “avoid” a green goal (as in Figure 6, bottom right). When a target headed toward a red goal to “avoid” a green goal (as in Figure 6, top center), the corresponding cell received a double minus (– –). Finally, when a target was heading toward a no-goal area to “avoid” another no-goal area (as in Figure 6, top right), this was assigned a zero (0). Note that the green/green and red/red combinations are not possible with this paradigm, because there is always only one green goal and one red goal. Using this classification, we could determine whether there was an effect of valence of the combined situation (area reached plus area avoided). Additionally, by comparing to baseline (0), it can be made clear whether a positive outcome gives enhanced attention or a negative outcome gives suppressed attention.

**Table 4.** Classification of reward/punishment for different combinations of (no-)goal area reached or avoided.

		Reached		
		Green	No goal	Red
Avoided	Green	–	Neg (−)	Neg (− −)
	No goal	Pos (+)	Pos/neg (0)	Neg (−)
	Red	Pos (++)	Pos (+)	–

Note that the green/green and red/red combinations are not possible with this paradigm, because there is always only one green goal and one red goal.

Table 5 shows mean detection rates for the categories introduced in Table 4. We pooled the mildly positive (+) combinations together, as well as the mildly negative (–) combinations, and performed a one-way ANOVA with five levels (– –, –, 0, +, ++). Because Mauchly's test indicated that the assumption of sphericity had been violated for the main effect of valence, χ^2^(9) = 48.96, *p* < 0.001, degrees of freedom were corrected using Greenhouse–Geisser estimates of sphericity (ε = 0.45). There was a significant effect of valence, *F*(1.78, 39.15) = 6.19, *p* = 0.006, η^2^ = 0.220, indicating that there was a difference in probe detection between the different levels. At closer inspection, comparing each level with the next (i.e., comparing – – with –, – with 0, 0 with +, and + with ++), there was only a significant difference between – and 0 (*p* < 0.001).

**Table 5.** Probe detection rates for different combination of (no-)goal area reached or avoided. Mean probe detection rates are expressed as percent (*SE*) based on 23 participants.

		Reached		
		Green	No goal	Red
Avoided	Green	—	16.3 (4.4)	16.4 (4.6)
	No goal	24.6 (3.1)	26.8 (3.0)	13.8 (2.9)
	Red	27.1 (3.1)	25.7 (3.5)	—

### Discussion

In this third experiment, we presented a different approach to an interactive MOT task than the one used in Experiments 1 and 2. Importantly, objects were now able to move through the central vertical wall, as well as bounce off. We found a main effect of move type (linear or bounce) in the current experiment, with higher probe detection rates when the object bounced off the obstacle wall than when it went through. This effect was also found by Atsma et al. (2012) when only one target needed to be tracked, but not during multiple object tracking (with three targets). This effect may be caused by the uncertainty that is introduced with the change in direction after a bounce. Viewers might need to perform an extra check or stay on the object longer to update its new trajectory when a bounce is expected, whereas this would not be necessary when its trajectory remains the same (i.e., when the object moves through the wall). In a future study, eye-tracking data might shed further light on this.

Another advantage of the current paradigm is the addition of a negative (red) goal. This not only makes it possible to compare the effects of reward and punishment on attention allocation but also provides a kind of baseline measurement from interactions with the no-goal areas. As expected, probe detection was significantly lower when an object moved toward, and eventually hit, a red goal. What's more, we saw a clear divide between negative valence (– and – –) on the one hand and neutral and positive valence (0, +, and ++) on the other hand. With the 0 level (neutral) acting as baseline, this is an indication that probe detection is suppressed in a negative (punishment) situation rather than enhanced in a positive (reward) situation (see below).

## General discussion

We investigated the effect that the presence of a goal might have on the spatial distribution of attention during MOT, specifically upon the moment a tracked object interacts with a wall (i.e., bounces off or passes through). We conducted three experiments using different forms of interactive MOT, where participants had to make tracked objects hit a goal object or area as often as possible by manipulating part of the display. In Experiment 1, participants could move a goal bar along one of the horizontal edges of the screen in order to catch the tracked target objects. In Experiment 2, the movement of the goal was restricted to only one-half of that horizontal edge. In Experiment 3, participants manipulated the permeability of a vertical wall that divided the screen in order to make the objects pass through or bounce off to either reach a green (positive reward) goal or avoid a red (negative reward) goal.

In Experiment 1, we saw an overall increase in probe detection on the side of the screen where the goal was situated. There did not appear to be any dissociation between the type of probe, Bounce or Linear, but for both cases there was an enhancement of attentional focus when the goal was in sight. In Experiment 2, we replicated the attentional enhancement for probes presented in the direction of the goal, with a higher resolution than in Experiment 1 by restricting the goal area. Additionally, we found a significant advantage for probes presented along the actual future path of the object (i.e., Bounce probes), indicating anticipatory attentional allocation. With Experiment 3, we found results similar to those of Experiments 1 and 2, as we also found lower probe detection when the target avoided a green goal to subsequently hit a no-goal area, compared with when the target avoided a no-goal area to subsequently hit the green goal. From this result alone, one might assume that the upcoming impact with the goal enhances the allocation of attention to the tracked object. However, when we increased the range of possible scenarios by introducing a negative reward in Experiment 3, turning the “no-goal” event into a neutral baseline, we saw that it is not so much *enhancement* of attention toward a (positive) goal but rather *suppression* of attention toward an area of negative reward.

A similar suppression effect in MOT was described by Pylyshyn (2006) on non-target objects, or what we refer to as distractors in the current study. Using a dot probe technique similar to the one we used here, with open space probes serving as a baseline, he found that non-targets were suppressed compared to target objects during MOT. A more recent study by Renton et al. (2019) even showed attentional suppression of avoidance-relevant items, both in a concurrent approach–avoidance task and when comparing attentional selection toward task-relevant objects between separate approach and avoidance tasks. In principle, non-target objects and non-goal-related areas (e.g., the no-goal side in Experiment 1) share the feature of not being directly relevant for the task, and attending to them would be unnecessary and possibly detrimental. Moreover, suppressing irrelevant elements of the display might even optimize performance on the task. However, when a situation should be actively avoided (for example, a target reaching the red goal in Experiment 3), we might expect to see less suppression or even enhancement around this area, as it is an area of interest. Instead, we saw suppression, especially when the outcome of an event was negative. This can be explained by the timing of the probe—at the moment the object touches the wall, when a mistake can no longer be corrected—and by the fact that there are two targets to track; that is, the participant may have already moved on to a situation they can still control. It is important to note here that participants tended to prioritize scoring points over detecting probes, so their main concern was to make sure the objects hit the green goal and avoided the red goal. We should also point out here that participants were not aware of the timing of the probes.

The current study shows that event valence, in the form of achieved reward or avoided punishment, on the one hand, and missed reward or achieved punishment, on the other hand, influences the allocation of attention during object tracking. Although we do not know exactly how positively our participants experienced the given reward or how negatively they experienced the punishment used, the paradigm used in Experiment 3 enabled us to define a ranking with increasing levels of valence that can be compared. Our findings are in line with previous studies investigating the effects of reward on attentional control (Anderson, Laurent, & Yantis, 2011; Hickey, Chelazzi, & Theeuwes, 2010), and complement our previous findings on anticipatory allocation of attention during multiple object tracking (Atsma et al., 2012; Frielink-Loing et al., 2017; Frielink-Loing et al., 2022).
